# Supplementary figures and images for: The ultrastructural characteristics of bile canaliculus in porcine liver donated after cardiac death and machine perfusion preservation
Source: PLoS One. 2020 May 29;15(5):e0233917. doi: 10.1371/journal.pone.0233917 (PMC7259665; doi:10.1371/journal.pone.0233917)

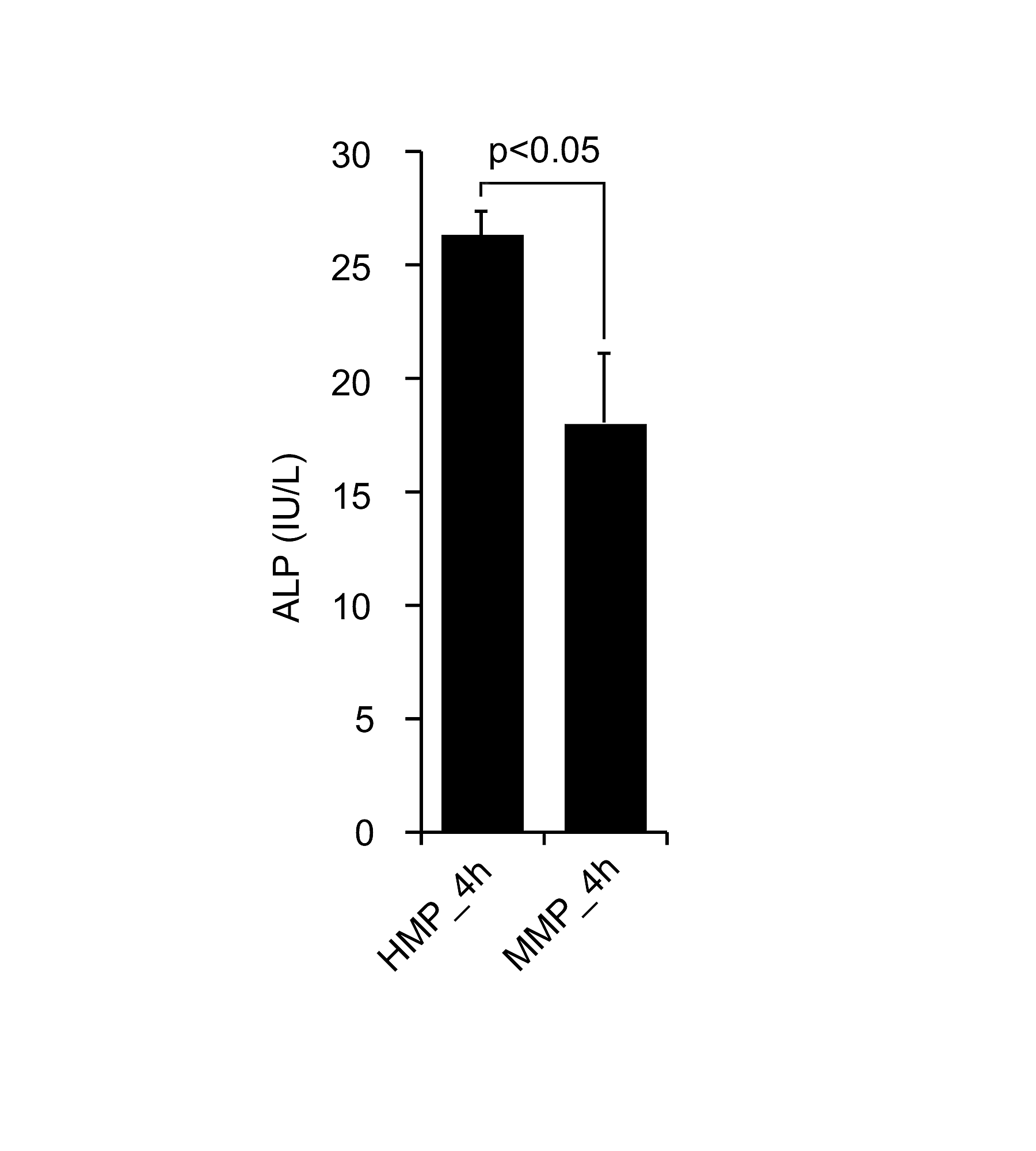

Supplement: S1 Fig — The levels of alkaline phosphatase (ALP) in the perfusate at 4 hours after hypothermic and midthermic machine perfusion preservation. Data are shown as the mean ± SEM. Unpaired two-tailed t-tests were used (p<0.05). (TIF) [file pone.0233917.s001.tif]
