# Supplementary material for: The ultrastructural characteristics of bile canaliculus in porcine liver donated after cardiac death and machine perfusion preservation
Source: PLoS One. 2020 May 29;15(5):e0233917. doi: 10.1371/journal.pone.0233917 (PMC7259665; doi:10.1371/journal.pone.0233917)
Supplement: Attachment — Submitted filename: Response to Reviewers R1.docx [file pone.0233917.s002.docx]

**RESPONSE TO REVIEWERS**

**of “The ultrastructural characteristics of bile canaliculus in porcine liver donated after cardiac death and machine perfusion preservation”**

**by Yo Ishihara, Hiroki Bochimoto, Daisuke Kondoh, Hiromichi Obara, Naoto Matsuno; ms. no PONE-D-19-34589**

**April 6, 2020**

On behalf of my co-authors, I would like to express our gratitude to the reviewers for their thoughtful review of the manuscript. They raise important issues and their comments are very helpful for improving the manuscript. We agree with almost all their comments and we have revised our manuscript carefully.

We are including all reviewers’ suggestions and clarifying the text when needed. We are confident that the new version of the manuscript will be greatly improved.

We respond below in detail to each of the reviewers’ comments. In addition, we include how we have revised things, or if we have slightly disagreed with something, we stated why. We hope that the reviewers will find our responses to their comments satisfactory, and we would be glad to respond to any further suggestion and comments that reviewers may have.

Please, find below the reviewers’ comments repeated in italics and our responses inserted after each comment.

We fully understand the COVID-19 pandemic is now having a big impact on you, but we look forward to hearing from you at your earliest convenience.

Sincerely,

Yo Ishihara M.D. and Hiroki Bochimoto M.D., Ph.D.

***Response to comments from Reviewer #1***

We are grateful to reviewer #1 for the critical comments and useful suggestions that have helped us to improve our paper considerably. As indicated in the responses that follow, we have taken all these comments and suggestions into account in the revised version of our paper.

***General comment***

*Thank you for asking me to review this paper by Ishihara et al. I am afraid I have a number of issues with the paper and experimental conclusions. In addition, or possibly explaining a number of my concerns, is a problem with the use of the English language.*

**Response:**

We really appreciate your thoughtful comments that helped improve the manuscript. We follow the advice in your specific comments described below to modify the section in our manuscript.

***Comment 1.***

*Abstract and throughout paper: “Warm MP.” The authors use this term but it is not an accepted term in the field. What they are actually describing is controlled oxygenated reperfusion, as described by Thomas Minor’s group; the temperature does not go above 22°C.*

**Response:**

We appreciate the reviewer's useful suggestion. We agree with the reviewer that we need to use more appropriate terminology. Nomenclature currently used for the warm types of liver machine perfusion is "warm perfusion" (Karangwa et al. 2016). In addition, we agree that our "warm MP" is similar to the "controlled oxygenated rewarming" described by Thomas Minor’s group. In addition, we think that our “warm MP” should be classified as “midthermic machine perfusion” according to the standardized nomenclature guidelines proposed by Karangawa et al (2016). Therefore, we added the sentence “midthermic MP (MMP), one type of warm perfusion [Karangwa et al. 2016]” to revised version (Line 68-69). Furthermore, the term “warm MP” was revised as "warm perfusion" or “midthermic machine perfusion” (abbreviated “MMP”) throughout this revised manuscript and figures.

Karangwa SA, Dutkowski P, Fontes P, Friend PJ, Guarrera J V., Markmann JF, et al. Machine Perfusion of Donor Livers for Transplantation: A Proposal for Standardized Nomenclature and Reporting Guidelines. Am J Transplant. 2016;1967: 2932–2942. doi:10.1111/ajt.13843

***Comment 2. Introduction. References***

*The authors include many references in the paper, but many are completely unrelated to the point they are trying to make. So, for example, references 4 and 5 do not discuss PNF and IRI post transplant; references 6 and 7 include one pig study and one NMP study of 30 patients compared to an historical control which do not address chronic rejection. A reference needs to be pertinent to the point being made, or it should not be there. I have not examined the other references in detail but I imagine similar issues pertain.*

**Response:**

We thank you for this critical comment. As the reviewer indicated, these citations might not be suitable for this manuscript. We carefully checked again the appropriate citations and its points, and replace the references 4-7 in original manuscript with more appropriate papers (Chan et al. 2008, Foley et al. 2005). In addition, some references that was cited in the original version have been removed from this revised version.

Chan EY, Olson LC, Kisthard JA, Perkins JD, Bakthavatsalam R, Halldorson JB, et al. Ischemic cholangiopathy following liver transplantation from donation after cardiac death donors. Liver Transpl. 2008;14: 604–10. doi:10.1002/lt.21361

Foley DP, Fernandez LA, Leverson G, Chin LT, Krieger N, Cooper JT, et al. Donation after cardiac death: the University of Wisconsin experience with liver transplantation. Ann Surg. 2005;242: 724–31. doi:10.1097/01.sla.0000186178.07110.92

***Comment*** ***3. Introduction.***

*“Recently, oxygenated cryogenic mechanical…”*

*Cryogenic is not the correct word – I suspect they mean hypothermic*

**Response:**

We completely agree with the reviewer. As the reviewer suggested, “hypothermic” is appropriate. Thus, we revised “oxygenated cryogenic mechanical perfusion” as “hypothermic MP (HMP)” in this version (Line 60).

***Comment*** ***4. Introduction.***

*“The WMP method more closely approximates physiological conditions by gradually increasing the perfusion temperature from the initial low temperature range”*

*There is nothing physiological in humans or pigs about being rewarmed from 8°C.*

**Response:**

We really appreciate the reviewer's critical comment. We completely agree with the reviewer that the sentence is inappropriate. We revised this sentence as “warm perfusion had introduced to maintain liver grafts at a more physiologic temperature compared with HMP to offers the opportunity to assess and possibly repair a metabolically active liver graft." in this version (Line 64-68).

***Comment 5. Introduction***

*“… deviating enzyme .”*

*What is meant by this?*

**Response:**

We really appreciate the reviewer's comment that make us realize the inappropriate sentence. We corrected the phrase “amount of deviating enzyme” as “hepatocellular enzyme release” in this revised version (Line 69-70).

***Comment*** ***6. Introduction.***

*“In addition, we confirmed that hepatocytes of WMP-treated liver grafts retain their function based on ultrastructural observations by scanning electron microscopy after osmium-maceration”*

*I am not sure how well you can infer that “liver grafts retain their function” from scanning EM*

**Response:**

We appreciate the reviewer's useful suggestion. This comment by the reviewer is completely correct. We previously confirmed the ultrastructure of hepatocytes, not the functions, by scanning electron microscopy. Thus, this sentence was revised as “we confirmed that hepatocytes of DCD liver grafts after MMP retain a functional ultrastructure compared to HMP, by using the observation method of scanning electron microscopy after osmium-maceration” in this version (Line 70-75).

***Comment*** ***7. Introduction***

*“One of the important physiological functions of hepatocytes is the production and secretion of bile [62], and detailed studies by transmission electron microscopy (TEM) on the capillaries involved in bile are performed [63].”*

*The second half of the sentence does not appear to follow the first. What is meant here?*

**Response:**

We really appreciate the reviewer's critical comment. We completely agree with the reviewer that the second half of the sentence does not to follow the first. Thus, we deleted this second sentence from this revised version (Line 78-79).

***Comment*** ***8. Materials and methods / Animals/***

*“cross-bred Large-Yorkshire, Landrace, and Duroc pigs”*

*Large-Yorkshire pigs. I believe the breed can either be called a Yorkshire pig or a Large White pig or a Large White Yorkshire pig.*

**Response:**

We appreciate the reviewer's helpful comment. We corrected the term “Large-Yorkshire” as “Large White” in this revised version (Line 91).

***Comment*** ***9. Materials and methods / Machine perfusion preservation.***

*The authors have developed a machine that supplies the PV with perfusate that has not passed through an oxygenator, while the HA is supplied by oxygenated perfusate. This is not a feature of any hypothermic oxygenated protocols in current practice – HOPE perfuses the portal vein alone with oxygenated perfusate (and there is no HA perfusion) and D-HOPE perfuses both HA and PV with oxygenated perfusate. There is a limited amount of oxygen that can be dissolved in UW-MPS solution and in the clinical studies investigators have tried to optimize this, as opposed to deliberately dilute it down. At normothermia using a blood based perfusate I could understand the approach they have followed, but it seems illogical at these temperatures with UW-MPS as a perfusate.*

**Response:**

We really appreciate the reviewer's carefully comment. We completely agree with the reviewer that our circuit is mismatch to a feature of any hypothermic oxygenated protocols in current practice. Thanks to you, we are able to recognize our mistake of description of machine perfusion systems. We revised the sentence "Additionally, the HA perfusion line has an oxygenator in the circuit" as "Additionally, an oxygenator was installed in the the upstream of the circuits for the PV and HA" in this revised version (Line 105-108). Moreover, the schema in Figure 1 was also corrected.

***Comment*** ***10. Materials and methods / Preparation and preservation /***

*“peeling around the hepatic artery and portal vein were performed”*

*What is meant by “peeling”*

**Response:**

We appreciate the reviewer's useful suggestion. Here, the "peeling" is an inappropriate term. We revised “peeling around the hepatic artery and portal vein were performed” as "hepatic artery and portal vein were isolated" in this revised version (Line 126-128).

***Comment*** ***11. Materials and methods / Preparation and preservation /***

*“euro-Collins”*

*Why was Euro-Collins used for liver preservation; it was long since shown to be inadequate as a liver preservation solution. It would have been more appropriate to flush the livers with UW solution.*

**Response:**

We appreciate this important question. We completely agree with the reviewer that the UW solution is considered the optimal solution for liver preservation. However, there are concerns over the cost and viscosity of the solution, with potentially cause of biliary lesions due to inadequate perfusion and hepatic circulation. Thus, flushing with Euro-Collins that is less viscous before infusing the UW solution may wash the blood out of the graft, and facilitate that the UW can be distributed in the microcirculation level effectively. We corrected the term “euro-Collins” as “Euro-Collins” in this revised version (Line 130-131).

***Comment*** ***12. Materials and methods / Preparation and preservation /***

*“University of Wisconsin gluconate”*

*By this do the authors mean machine perfusion UW solution, or was it simple cold storage UW to which they added gluconate, in which case how much was added?*

**Response:**

We apologize that this part was not clear. Our modified University of Wisconsin gluconate solution consisted of sodium gluconate 17.5 g, KH2PO4 3.4 g, trehalose 10 g, glutathione 0.9 g, adenosine 1.3 g, HEPES 4.7 g, penicilline 200,000 U, dexamethasone 16 mg, MgSO4 1 g, caffeine 4 g, polyethylene glycol 10 g, and glycine 1 g per 1 L. We added the information in this version (Line 134-136).

***Comment*** ***13. Materials and methods / Preparation and preservation /***

*“The degree of bile duct dysfunction at four hours after MP…”*

*ALP will tell about cellular release of the enzyme, but not about bile duct function or dysfunction. I realize this is sematic, but it is important to appreciate what you are actually measuring; some readers may not be so discerning*

**Response:**

We appreciate the reviewer's helpful suggestion. We agree with the reviewer that the ALP cannot directly tell about bile duct dysfunction or dysfunction. Thus, we revised the phrase "bile duct dysfunction" as "biliary injury" in this version (Line 141).

***Comment*** ***14. Results. General comment***

*The results are studies of the liver biopsies after different periods of MP, with no reperfusion step. It would be much more interesting and clinically meaningful to see what the effects of the different perfusion interventions are post reperfusion.*

**Response:**

We agree with the reviewers that the analysis of the post reperfusion step is more interesting and clinically meaningful. However, we can focus on the basic morphological aspect of bile canaliculus only after machine perfusion because of the unavoidable limitation of animals and experimental systems in the present research. Thus, we acknowledged that present research has limitation concerned with clinical meaning. In addition, we added the sentence “In future study, more clinically appropriate MP conditions to preserve the functions of the liver grafts should be established by using normothermically reperfusion systems” to conclusion paragraph in this revised version (Line 379-381). Please see also the response to comment #5 of another reviewer.

***Comment*** ***15. Results. Figures.***

*The figure captions need to be with the figures not in the middle of the text*

**Response:**

We are terribly sorry, but the *PLOS ONE* guideline requires this format style that the figure captions are inserted in the middle of the text.

***Comment*** ***16. Results.***

*“These findings indicated that, MP preservation, especially WMP, of liver grafts after warm ischemia enabled the ultrastructure of the bile canaliculi to be maintained and restored, although these effects were insufficient for the recovery of bile production.”*

*In hypothermic or even at 22°C I would not expect bile production, particularly in the absence of a substrate.*

**Response:**

We thank you for this comment. We completely agree with the reviewer that bile production in the absence of a substrate under 22°C does not appear to occur. We deleted the latter half of this sentence (“although these effects were insufficient for the recovery of bile production”) in this version (Line 272-273). Please see also the response to comment #4 of another reviewer.

***Comment*** ***17. Discussion.***

*“Because the actual hepatocyte metabolism due to interaction with other organs cannot be reproduced [96], even monitoring of liver grafts during reperfusion using blood from the same individual after perfusion storage does not seem sufficient to evaluate the liver graft function.”*

*I disagree. Reperfusion at normothermia ex situ would further inform the degree to which the two preservation modalities affect liver histological appearance.*

**Response:**

We appreciate the reviewer's critical suggestion. We agree with the reviewer that the reperfusion at normothermia ex situ would further inform the degree to which the two preservation modalities affect liver histological appearance. Therefore, we deleted this sentence from this revised version, and added the sentence “In addition, this study did not evaluate the effect in reperfusion at normothermia” and the phrase “and reperfusion at normothermia ex situ” in this version (Line 361-362 and 367-368).

***Comment*** ***18.***

*I am not a histologist so cannot comment on the changes between the electron micrographs, but recommend an independent review of these*

**Response:**

Again, thank you for all of comments. We carefully responded to questions and indications from another reviewer, and thus please see also the response to another reviewer.

***Response to comments from Reviewer #2***

We are grateful to reviewer #2 for the critical comments and useful suggestions that have helped us to improve our paper considerably. As indicated in the responses that follow, we have taken all these comments and suggestions into account in the revised version of our paper.

***General comment***

*Ishihara et al have presented their work on ultrastructural characteristics of bile canaliculus in machine perfused porcine livers following donation after circulatory arrest. It’s an extension to their similar previous study on hepatocyte ultrastructure. The authors retrieved porcine livers after 1 hour of warm ischaemia and subjected grafts for four hours of machine perfusion, either hypothermic at 8 C or rewarmed to 22 C. They conclude that warm preservation is better in preserving and restoring the ultrastructure in bile canaliculus. I congratulate the authors on these important findings. In our experience, we are confident in predicting hepatocyte function in normothermic machine perfusion livers but cholangiopathy is still encountered in such grafts. Assessment of cholangiocyte function is still experimental and needs more investigation. My comments for this manuscript are as follows:*

**Response:**

We really appreciate the reviewer for having an interest in our research. The reviewer has a good understanding of the goal of our manuscript. The positive comments to our findings from the reviewer really encourage us.

***Comment*** ***1. Line 55, page 3.***

*Machine perfusion is a more accepted term and is what mentioned in the abstract and used elsewhere in the manuscript. I would suggest changing it to machine perfusion than 'mechanical perfusion'.*

**Response:**

We appreciate the reviewer's useful suggestion. We completely agree with the reviewer. The term “mechanical perfusion” was corrected as “machine perfusion” in this revised version (Line 56 and 60).

***Comment*** ***2. Line 96, 97, page 4.***

*Flow should be pulsatile for the hepatic artery and non-pulsatile for portal vein. It has been reported the other way around.*

**Response:**

We really appreciate the reviewer's helpful comment that make us realize the inappropriate description. We corrected the phrase “allowing non-pulsatile and pulsatile” as “allowing pulsatile and non-pulsatile” in this revised version (Line 104-105).

***Comment* *3. Line 114, page 5.***

*Induce cardiac arrest in place of induce to cardiac arrest.*

**Response:**

We appreciate the reviewer's useful suggestion. We completely agree with the reviewer. We corrected this point according to the reviewer’s suggestion in this version (Line 124).

***Comment*** ***4.***

*Bile production, as stated by the authors, was not seen after HMP or WMP. However, bile production is not seen in hypothermic liver perfusion even in clinically transplantable livers. Bile production is not a marker of viability in HMP livers because of low metabolic activity at low perfusion temperature.*

**Response:**

We appreciate the reviewer's useful suggestion. We completely agree with the reviewer that bile production is not a marker of viability in our series of machine perfusion of present research. Thus, we deleted the sentence (“although these effects were insufficient for the recovery of bile production”) in result section (Line 272-273), the whole paragraph (Line 327-331) and the sentence (“However, the present conditions could not resume bile production, and more appropriate MP conditions shuld therefore be established to preserve the functions of the liver grafts.”) in discussion section (Line 377-379) in revised version. Please see also the response to comment #16 of another reviewer.

***Comment* *5.***

*It would be interesting to find ultrastructural changes in normothermically perfused livers with blood-based perfusate and comparison with HMP and WMP. Also the structure of first and second-order bile ducts. It has been acknowledged by the authors in the discussion section and I would strongly recommend it.*

**Response:**

We appreciate the reviewers critical comment. We agree with the reviewer that the analysis of normothermically reperfusion and the structure of first and second-order bile ducts is more interesting. However, we can focus on the basic morphological aspect of bile canaliculus only after machine perfusion because of the unavoidable limitation of animals and experimental systems in the present research. Thus, we acknowledged that present research has limitation concerned with clinical meaning. In addition, we added the sentence “In future study, more clinically appropriate MP conditions to preserve the functions of the liver grafts should be established by using normothermically reperfusion systems” to conclusion paragraph in this revised version (Line 379-381). Please see also the response to comment #14 of another reviewer.

***Comment* *6.***

*Bile pH as a predictor of cholangiopathy needs validation in larger series and has been reported only by groups in studies that included few patients.*

**Response:**

We appreciate the reviewer's critical comment, and we completely agree with the reviewer that bile pH as a predictor of cholangiopathy needs validation in future. Thus, we deleted whole of the paragraph (Line 347-356) describing about bile pH as a predictor of cholangiopathy in this revised version.

***Comment* *7.***

*Lastly, now the wider consensus is to use the term ‘circulatory arrest’.*

**Response:**

We thank you for this helpful suggestion. We revised the term “cardiac arrest” as “circulatory arrest” throughout the manuscript.
